# Potential role of estradiol in ovariectomy-induced derangement of renal endocrine functions

**DOI:** 10.1080/0886022X.2019.1625787

**Published:** 2019-06-20

**Authors:** Ahmed A. El-Gendy, Wael M. Elsaed, Hesham I. Abdallah

**Affiliations:** a Department of Medical Physiology, Faculty of Medicine, Taibah University, Madinah, Saudi Arabia;; b Department of Medical Physiology, Faculty of Medicine, Mansoura University, Mansoura, Egypt;; c Department of Anatomy & Embryology, Faculty of Medicine, Taibah University, Madinah, Saudi Arabia;; d Department of Anatomy & Embryology, Faculty of Medicine, Mansoura University, Mansoura, Egypt;; e Department of Anatomy & Embryology, Faculty of Medicine, Ain Shams University, Cairo, Egypt

**Keywords:** Ovariectomy, estradiol, tamoxifen, renal endocrine, ER-β

## Abstract

Menopause is an important physiological event associated with structural and functional changes in the kidneys. An animal model of bilateral ovariectomy was used to study the effects of estrogen depletion, replacement and antiestrogen on renal structure and endocrine function. Sixty female rats were divided into six groups; group I was the control group, the remaining five groups underwent ovariectomy: group II received no treatment. The other groups received estradiol in group III, tamoxifen in group IV, estradiol followed by tamoxifen in group V and tamoxifen followed by estradiol in group VI. Serum creatinine, blood urea nitrogen, and endocrine functions of kidney were measured. Tissue samples were examined both microscopically for beta estrogen receptors and ultrastructurally for cell changes. Groups II, IV & VI showed a significant increase in creatinine, blood urea nitrogen, renal malondialdehyde, renal erythropoietin, plasma renin and plasma prostaglandin E2 and a significant decrease in renal antioxidants and serum vitamin D3. Groups III &V had a significant decrease in creatinine, blood urea nitrogen, renal malondialdehyde and renal erythropoietin with an increase in renal antioxidants, plasma prostaglandin E2 and serum vitamin D3. Histopathological and ultrastructural examinations revealed atrophic tubular changes in group II. The changes were less marked in groups III &V and more extensive in groups IV & VI. Estrogen receptor beta staining showed progressively increased expression in the absence of estrogen. Structural and most endocrine functions of the kidney were significantly affected by estradiol deficiency. Estradiol replacement exhibited a protective effect on renal tissue and endocrine functions.

## Introduction

Besides the excretory role of the kidney, it has various endocrine functions some of which are still being discovered and elucidated [1]. Several hormones with different functions are secreted by the kidneys. Renin, for example, is released from the juxtaglomerular cells and has autocrine and paracrine effects [2]. Angiotensin-converting enzyme presents in abundance in the proximal tubule (PT) brush border, apart from other sites in the renal tissue [3]. Erythropoietin (EPO) is produced in the interstitial cells of the renal cortex near the bases of the PT and peritubular cells in response to the sensing of oxygen deficiency [4]. In addition, the active forms of vitamin D (vitamin D3) is synthesized in the mitochondria of the PT [5,6]. Furthermore, the renal tissue is involved in the production of adrenomedullin, endothelins, and prostaglandins [7]. Prostaglandin E2 is an important physiologic regulator of blood flow and electrolytes and water homeostasis in the kidney [8]. Prostaglandin E2 is synthesized by cyclooxygenases; COX-1 and COX-2 which stimulate the formation of prostaglandins from arachidonic acid [9].

The kidney also plays an important role in inflammatory cytokines production and regulation through the control of renin-angiotensin system (RAS) cascade [10]. At the same time, kidneys are targeted by various hormones to regulate their functions especially sex hormones [11]. Estradiol (E2) is essential for the normal cellular growth and differentiation, maintenance of the kidney functions [12] and regulation of the renal homeostatic activities [13]. The disturbance in serum E2 level is supposed to affect the morphology and functions of the kidneys. The incidence of kidney diseases, for instance, is known to be gender-related [14]. Estradiol has a protective role against the progression of chronic renal diseases [15]. While decreased synthesis of E2 makes the kidney more vulnerable to renal oxidative injuries [16]. On the other hand, it has been observed that the E2 replacement therapy modifies renal dysfunction and inhibits ultrastructural deterioration after bilateral ovariectomy (OVX) [17,18].

Ovariectomy in experimental animals is a considerable model of studying the effects of E2 deficiency in menopause [19]. Removal of the ovaries accelerates the progression of the renal pathological changes, increases the oxidative stress by the high production of reactive oxygen species (ROS), and disturbs the renal functions involving endocrines [20].

Estradiol mediates its bioeffect by binding to estradiol receptors (ERs) [21]. ERs are of 3 subtypes; ER-α, ER-β and G-protein coupled receptor [22,23]. Although the structures of ER-α and ER-β are of similar nature, their tissue distributions and biological functions are different [24]. ER-α receptors were the only identified receptors until 1996. Estradiol depletion resulted in an imbalance in ER-α and ER-β expression [25]. The ER-β receptors in the kidney play an important role in improving renal performance [12]. ER-β have been localized in the smooth muscles of the renal blood vessels and have a crucial role in response to vascular injury in pre- and post-menopausal women [25]. Moreover, ER-β receptors have been linked to carcinogenesis and apoptosis of the renal tissue [21, 26]. ER-β agonists were found to improve the activities of antioxidant enzymes in the renal tissue [27]. However, the involvement of ER-β in mediating E2 actions and its distribution in the renal tissue is still in need for more investigations.

A known modulator of the ERs is tamoxifen (TAM), which acts by binding to the ERs countering E2 effects on the cells [28]. This action favors its use in the treatment of hormone-dependent breast cancer [28]. Despite this important anticarcinogenic effect, TAM produces nephrotoxicity with overproduction of ROS and induction of oxidative stress [29]. The exact role of E2 therapy after TAM treatment for its protective effect against TAM-induced renal injury is not known. While the effects of TAM on kidney ER-α have been studied thoroughly [30], however, its effect on ER-β is still deficient in literature.

Estradiol administration in experimental OVX and TAM-induced nephrotoxicity provides an appropriate experimental model for studying the endocrine function, morphological and ultrastructural changes in the kidneys induced by menopause with the involvement of the ER-β receptors.

## Materials and methods

### Chemicals

Estradiol was supplied as Folone ampoules 1 mL (Misr Co., for Pharm. ind. S.A.E. Cairo, Egypt). Each ampoule contains 5 mg estradiol benzoate. Estradiol was injected at a dose of 250 µg/kg/day, s.c. [31].

Tamoxifen (Sigma Chemical Co., St Louis, MO, USA) was suspended in 100 μL ethanol and dissolved in 900 μL corn oil. It was administered at a dose of 45 mg/kg/day intraperitoneally [30].

### Experimental protocol

Sixty adult female Sprague–Dawley rats, 5 ± 0.4 weeks of age, weighing 165 ± 15 g each, were housed (Animal House, Medical Physiology department, Faculty of Medicine, Mansoura University, Egypt) in standard conditions under approved procedures by Medical Research Ethical Committee of Mansoura University, Egypt (IRB).

The rats were randomly assigned into six equal groups of 10 animals; one control group (Group I) and five OVX study groups. All remaining fifty rats were subjected for OVX under anesthesia (ketamine, 75 mg/kg, i.p. and xylazine, 10 mg/kg, i.p.) following the usual sterile surgical procedures [32].

### Animal grouping and sample collection

**Figure 1.**
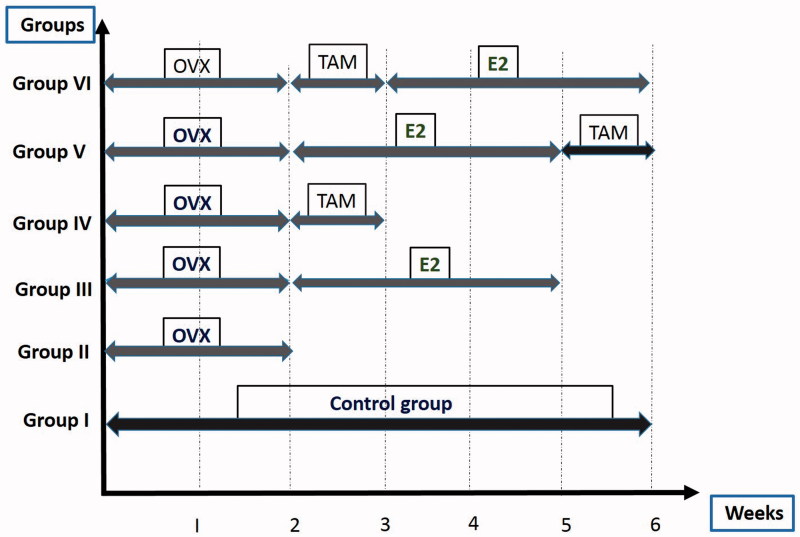
Diagram of schematic representation of the study protocol of the rat groups in weeks; Group I is the control group, group II is the ovariectomy (OVX) group, group III is the estradiol (E2), group IV is tamoxifen (TAM), group V is E2 followed by TAM and group VI is the TAM followed by E2 treatment.

The control group (group I) did not undergo any treatment. The remaining experimental 5 groups were further subdivided according to the treatment given after OVX. Group II received no treatment. After 2 weeks of OVX. Estradiol was given for 3 weeks in group III, TAM for 7 days in group IV and TAM (for 7 days) followed by E2 (for 3 weeks) in group V, and E2 (for 3 weeks) followed by TAM (for 7 days) in group VI. A schematic representation of the timing of drug treatments is illustrated in Figure 1.

At the end of the experimental periods, overnight fasted rats were anesthetized by pentobarbital (60 mg/kg). Blood samples were collected by puncture of rat tail artery with a 25-gauge needle and collected into two tubes: the first one contained EDTA and was centrifuged at 2,000 rpm(1000 *g*) for 1 min at 4 °C to obtain plasma samples which were used for measurement of renin, PGE2 and E2. In the second tube, the blood sample was collected without anticoagulant to obtain serum for determination of serum creatinine (SCr), blood urea nitrogen (BUN) & serum vitamin D (SD).

The animals were sacrificed under the anesthesia and the kidneys were immediately excised from the dissected animals, trimmed of fats, and washed with physiological saline solution [33]. One excised kidney was used for the preparation of renal tissue homogenate by homogenizing 1 g of the kidney tissue in 10 mL potassium phosphate buffer containing 0.1 mM EDTA and centrifugation at 3000 rpm for 15 min.

### Assessment of kidney function biomarkers, oxidative stress parameters, and serum E2

Serum creatinine was assessed using the Jaffépicric acid procedure, with Sigma kit 555-A # (Sigma–Aldrich Chemical Co.) [34]. Blood urea nitrogen was assessed by the enzymatic method (modified Berthelot reaction) (dp international; Tuscaloosa, USA) [35] and serum vitamin D (SD) was assessed using the ELISA [36].

Plasma renin (PR) was measured by an automated immunochemiluminometric assay [37]. Plasma Prostaglandin E2 (PGE2) was estimated by using titerzyme PGE2 enzyme immunoassay kit, manufactured by Perspective Biosystems [38]. Estradiol was measured using an E2 DSL-4400 Radioimmunoassay kit (Diagnostic Systems Laboratories, Inc., Webster, TX, USA) according to the manufacturer’s instructions [39].

Renal erythropoitin (EPO) was determined from the homogenates by EPO ELISA kit [40]. The level of renal Malondialdehyde (MDA) as a marker of lipid peroxidation (LPO) [41] was determined by thiobarbituric acid 0.67% and trichloroacetic acid 10% method [42]. The activity of renal Glutathione Peroxidase (GPx) [43], Catalase (CAT) [44] and Superoxide Dismutase (SOD) were measured as previously described by Afrazeh et al. [45].

### Microscopic study

The other excised kidney was divided longitudinally into 2 halves; one half was fixed in 10% formalin in water for one week, dehydrated in ascending grades of ethanol, cleared in xylol and embedded in paraffin. Paraffin sections (4–5 μm thick) were cut and stained with hematoxylin and eosin [46] and ER-β immune staining [47]. The sections were examined with an Olympus light microscope and photographed.

### Ultrastructural study

The other half of the kidney was cut into 1 mm^2^ pieces and were immediately dipped in 2.5% glutaraldehyde and kept in the refrigerator at 4 °C for 2 h and processed for examination with PEM-100 transmission electron microscope [48] at EM unit in Mansoura University.

### Morphometric study

The area of immunopositivity of ER-β staining was measured under 400X magnification. The slides were digitized and saved as TIFF files. The results obtained from the images were analyzed on IntelR Core I3R built computer by using Video Test Morphology R software (Saint-Petersburg, Russia) with a particular built-in routine for calibrated immune staining quantification in relation to the total sectional area [49].

Five rats died during the surgical procedures probably from unintended hemorrhage. The final numbers were, group I = 10, group II = 9, group III = 9, group IV = 8, group V = 10, and group VI = 9 animals.

### Statistical analysis

Data are expressed as mean ± standard deviation. Statistical analysis was performed using statistical package for social science (SPSS software, version 22). Significance between the different experimental groups was determined by one-way ANOVA, followed *post hoc* test for multiple comparisons using the least significant difference. The significance is acceptable at *p* < 0.05.

## Results

### Assessment of kidney function biomarkers, oxidative stress, antioxidants parameters and serum E2

Serum creatinine, BUN and MDA were significantly increased in group II (OVX group) in comparison to the control group (group I) (Table 1). As a result of E2 administration after OVX in group III, there was a significant decrease in their levels in comparison to group II. Wherever there was a significant increase of SCr and BUN in group IV due to the effect of TAM after OVX in comparison to group II whereas, E2 given before TAM in group V caused a significant decrease in comparison to group IV. At the same time, TAM administered before E2 in group VI caused a significant decrease in comparison to group V. The antioxidants (GPx, CAT and SOD) were significantly decreased in group II in comparison to group I. There was a significant increase in their levels in group III in comparison to group II. In addition, there was a significant decrease in antioxidants in group IV in comparison to group II. Group V showed a significant increase in comparison to group IV, at the same time, CAT & SOD in group VI showed a significant decrease in comparison to group V but no significant change in GPx levels was reported.

**Table 1.** Effect of ovariectomy, estradiol and tamoxifen on serum creatinine (SCr) (mg/dl), blood urea nitrogen (BUN; mg/dl), renal oxidative stress (MDA) antioxidants parameters (GPx, CAT, SOD), renal erythropoietin (EPO pg/ml), plasma renin (PR mU/L), plasma prostaglandine E2 (PGE2 pg/ml), serum vitamin D (SD pmol/l) and plasma 17 b-estradiol levels (E2 pg/ml) (mean ± SD).

Parameters	Group 1 *N* = 10	Group II *N* = 9	Group III *N* = 9	Group IV *N* = 8	Group V *N* = 10	Group VI *N* = 9
SCr (mg/dl)	0.4 ± 0.05	3.5 ± 0.21 *p*1 < 0.001	1.7 ± 0.31 *p*2 < 0.001	5.3 ± 0.14 *p*3 < 0.01	3.4 ± 0.13 *p*4 < 0.01	2.5 ± 0.14 *p*5 < 0.01
BUN (mg/dl)	36.3 ± 0.3	66.5 ± 1.3 *p*1 < 0.001	56.4 ± 2.1 *p*2 < 0.001	87.4 ± 1.2 *p*3 < 0.01	60.1 ± 1.2 *p*4 < 0.01	51.8 ± 0.9 *p*5 < 0.01
Renal MDA (nmol/g protein)	63.4 ± 4.93	90.30 ± 5.34 *p*1 < 0.001	70.34 ± 5.32 *p*2 < 0.01	116.2 ± 9.51 *p*3 < 0.001	100.34 ± 8.35 *p*4 < 0.01	80.35 ± 7.31 *p*5 < 0.01
Renal GPx (U/L)	46.7 ± 3.11	30.34 ± 2.41 *p*1 < 0.001	35.34 ± 3.46 *p*2 < 0.01	19.2 ± 3.13 *p*3 < 0.01	25.35 ± 2.87 *p*4 < 0.001	22.35 ± 3.12 *p*5:ns
Renal CAT (U/L)	34.6 ± 2.18	27.37 ± 1.85 *p*1 < 0.01	30.37 ± 1.96 *p*2 < 0.05	14.4 ± 1.83 *p*3 < 0.001	24.35 ± 2.10 *p*4 < 0.001	20.83 ± 2.31 *p*5 < 0.05
Renal SOD (U/L)	56.1 ± 2.56	30.24 ± 2.34 *p*1 < 0.001	47.53 ± 2.76 *p*2 < 0.001	22.1 ± 3.15 *p*3 < 0.001	31.35 ± 3.52 *p*4 < 0.01	27.20 ± 2.12 *p*5 < 0.05
Renal EPO (pg/ml)	44.3 ± 1.8	69.5 + 1.7 *p*1 < 0.001	26.7 ± 1.2 *p*2 < 0.001	76.2 ± 1.0 *p*3 < 0.05	40.8 ± 1.8 *p*4 < 0.001	41.9 ± 1.1 *p*5:ns
PR (mU/L)	200.5 ± 32.3	240.1 ± 18.3 *p*1 < 0.001	101.3 ± 20.2 *p*2 < 0.001	270.4 ± 13.8 *p*3 < 0.01	225.5 ± 12.5 *p*4:ns	230.5 ± 35.2 *p*5:ns
PGE2 (pg/ml)	357.23 ± 12.43	2073.42 ± 283.65 *p*1 < 0.001	2382.12 ± 127.21 *p*2 < 0.001	2794.76 ± 379.56 *p*3 < 0.001	2960.53 ± 132.41 *p*4 < 0.001	2890.34 ± 201.23 *p*5:ns
SD (pmol/l)	200.5 ± 32.3	101.3 ± 20.2 *p*1 < 0.001	149.1 ± 18.3 *p*2 < 0.001	80.4 ± 13.8 *p*3 < 0.05	160.5 ± 12.5 *p*4 < 0.01	85.5 ± 35.2 *p*5 < 0.001
PlasmaE2 (pg/ml)	35.5 ± 4.9	8.5 ± 1.4 *p*1 < 0.001	20.8 ± 2.4 *p*2 < 0.001	9.6 ± 1.2 *p*3 < 0.05	21.3 ± 2.7 *p*4 < 0.001	25.2 ± 2.4 *p*5 < 0.05

*p*1: statistical significance between control group I versus group II.

*p*2: statistical significance between group II versus groupIII.

*p*3: statistical significance between group II versus group IV.

*p*4: statistical significance between group IV versus group V.

*p*5: statistical significance between group V versus groupVI.

Test of significance *p* < 0.05.

ns: Not significant; SD: Standard deviation.

Renal erythropoietin was significantly increased in group II in comparison to group I. There was a significant decrease in group III in comparison to group II. Moreover, there was a significant increase in group IV in comparison to group II, at the same time, group V showed a significant decrease in comparison with group IV but group VI showed no significant change when compared to group V.

PR was significantly increased in group II in comparison to group I. In group III, there was a significant decrease in comparison to group II. A significant increase in group IV in comparison to group II was noted. No significant change when comparing group V to group IV and group VI to group V was reported.

PGE2 was significantly increased in group II in comparison to group I. In group III, there was also a significant increase in comparison to group II. A significant increase of PGE2 in group IV in comparison to group II was recorded. Group V showed a significant increase as a comparison with group IV, but group VI showed no significant change in comparison to group V.

Serum vitamin D3 was significantly decreased in group II in comparison to group I. Group III, had a significant increase in comparison to group II. Wherever there was a significant decrease in group IV in comparison to group II. Group V showed a significant increase in comparison with group IV, but group VI showed a significant decrease in comparison to group V.

Plasma E2 level was significantly decreased in group II in comparison to group I. Group III also showed a significant increase in comparison to group II. There was a significant increase in group IV in comparison to group II. A significant increase in comparing group V to group IV and group VI to group V was reported.

### Light microscopy

**Figure 2.**
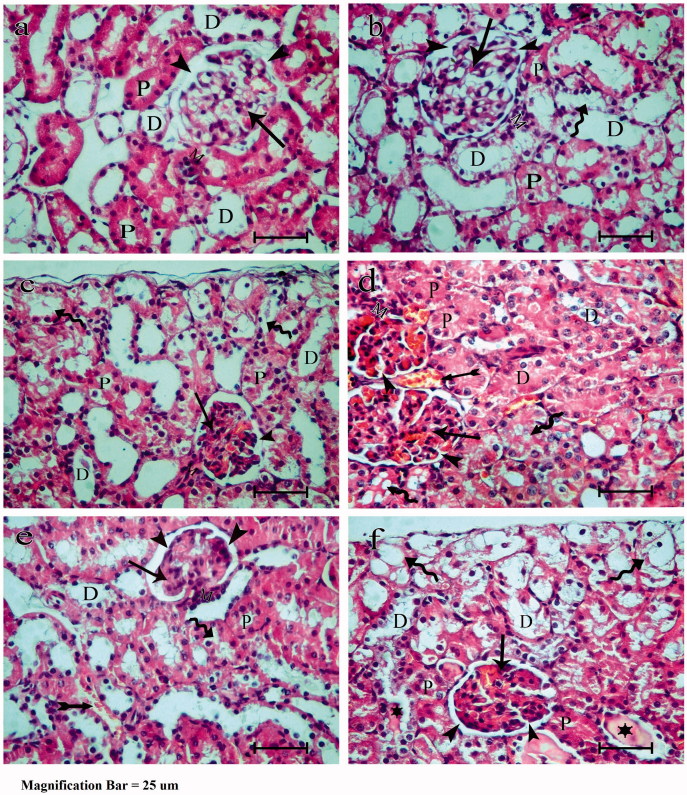
Photomicrograph of hematoxylin and eosin stained paraffin sections of rat renal cortex showing glomerulus (arrow) of the control group (group I) (a), dilated glomerular capillaries in ovarictomized group (group II) (b). Congested glomerular capillaries in estradiol treated group (group III) (c), segmented glomerulus with dilated congested capillaries in tamoxifen treated groups IV (d) and VI (f) and glomerular capillaries moderately dilated and congested in estradiol and tamoxifen treated group (group V) (e). Bowman’s space (arrow-head) is apparently narrow in groups II, IV and VI and apparently normal in groups III and V. PT (P) is lined with cuboidal cells with acidophilic cytoplasm, rounded open face nuclei and apical brush border in group I and lost apical brush border and weak eosinophilic vacuolated cytoplasm (curved arrows) in groups II, IV, V and VI. Some cells have apical brush border with some cytoplasmic vacculations (curved arrow) in group III. DT (D) with large clear lumen and absent brush border in groups I and III, have pale vacuolated cytoplasm with widened cavity in group II but have pale vacuolated cytoplasm with widened cavity and congested intervening vasculature (tailed arrow) in groups IV and V. The DT appears widened with pale vacuolated cell lining and eosinophilic cast in some of the lumens (stars). Most of the outer cortical tubules appeared vacuolated (curved arrow) in group VI. The macula densa (M) appears at the vascular pole of the glomerulus in groups I, II and III and is deeply stained in groups IV and V.

Microscopic examinations of the control group revealed normal renal corpuscles with typical proximal tubules (PT) and distal tubules (DT) (Figure 2(a)). The sections from group II showed apparent congested glomerular capillaries and narrow Bowman’s space. The PT had vacuolated cytoplasm with loss of the apical brush border in most of the lining cells. The DT had pale vacuolated cytoplasm with widened tubular cavities and congested intervening vasculature (Figure 2(b)). Estradiol-treated group III showed apparent normal Bowman’s space with less evident congestion of the glomerular capillaries. The PT retain the apical brush border with few cytoplasmic vacuolations. The DT appeared almost normal (Figure 2(c)). In group IV, following TAM treatment showed seemingly narrow Bowman’s space with segmented glomerulus and congested capillaries. The PT and DT were more or less similar to group II apart from the congested vasculature (Figure 2(d)). Group V (E2 + TAM group) revealed apparent dilated and congested glomerular capillaries. Some of the cell lining of the PT lost the apical brush border and had vacuolated cytoplasm. Few of the DT epithelial cells had pale vacuolated cytoplasm with widened cavities and congested blood vessels (Figure 2(e)). When TAM preceded E2 in group VI, glomeruli and PT were more or less similar to that of group II. The DT had a pale vacuolated cell lining with a widened cavity and precipitated eosinophilic cast in the lumen of some. Most of the outer cortical tubules appeared vacuolated (Figure 2(f)).

### Er-β immune staining

**Figure 3.**
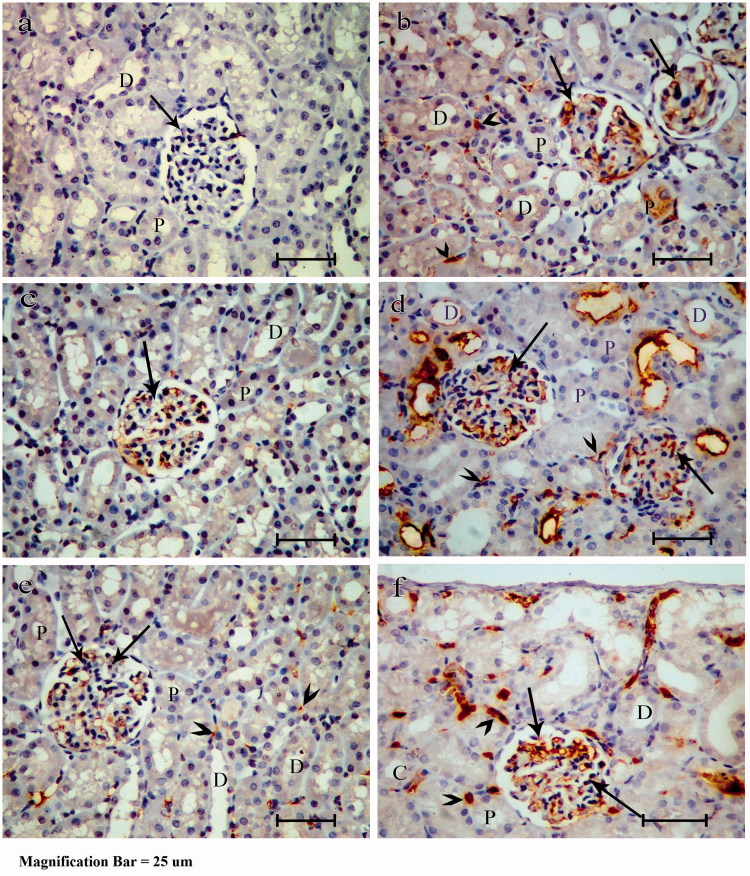
Photomicrograph of Er-β immune stained paraffin sections showing almost absent expression of the stain in the cytoplasm of glomerular cells (arrow) in the control group (group I) (a), moderate expression in the cytoplasm of glomerular cells (arrows) in ovariectomized group (group II) (b) and group estradiol treated group (group V) (e), weak expression in the glomerular cells’ cytoplasm (arrow) in group estradiol treated group (group III) (c), marked expression in the glomerulus cells’ cytoplasm (arrows) in group tamoxifen treated group (group IV) (d) and group tamoxifen and estradiol treated group (group VI) (f). Complete absent expression of the stain in the interstitial cells and proximal convoluted tubules (P) in groups I and III, few expression in some of the interstitial cells (arrow heads) and PT (P) in groups II and V and few expression in some of the DT (D) in group V. Marked expression in the interstitial cells (arrow-heads), PT (P) and DT epithelial cells (D) in groups IV and VI and no expression in the DT (D) in groups I, II and III is noted.

Group I stained sections showed almost absent expression of the stain in the glomerular cells’ cytoplasm and complete lack of expression in the interstitial cells, PT and DT epithelial cells (Figure 3(a)). Group II showed moderate expression in the glomerular cells’ cytoplasm, little expression in some of the PT, interstitial cells, and no expression in the DT (Figure 3(b)). Group III had a weak expression in the glomerular cells’ cytoplasm, almost absent expression in the interstitial cells, PT and DT epithelial cells (Figure 3(c)). Group IV showed marked expression in the glomerulus cells’ cytoplasm, PT, interstitial cells, and DT (Figure 3(d)). Group V showed moderate expression in the glomerulus cells’ cytoplasm, weak expressions in some of the PT, interstitial cells, and DT epithelial cells (Figure 3(e)). Group VI showed more expression of the Er-β antibody in relation to group V (Figure 3(f)).

Quantitative measurement of the area of immunopositivity showed significantly decreased in group II in comparison to group I. Group III, had a significant increase in comparison to group II. However, there was a significant decrease in group IV in comparison to group II. Group V showed a significant increase in comparison with group IV, but group VI showed a significant decrease in comparison to group V (Table 2).

**Table 2.** The Relative ER-β positive immuno-staining area expressed as the percentage of the total kidney sectional area, in ER- β immune stained sections. Data are the mean ± SD.

Parameters	Group I *N* = 10	Group II *N* = 9	Group III *N* = 9	Group IV *N* = 8	Group V*N* = 10	Group VI *N* = 9
ER-β immune staining relative area (μm^2^)	11.17 ± 0.32	14.31 ± 2.32 *p*1 < 0.05	41.16 ± 5.72 *p*2 < 0.001	23.54 ± 8.67 *p*3 < 0.001	10.26 ± 4.47 *p*4 < 0.001	21.12 ± 8.12 *p*5 < 0.001

*p*1: statistical significance between control group I versus group II.

*p*2: statistical significance between group II versus group III.

*p*3: statistical significance between group II versus group IV.

*p*4: statistical significance between group IV versus group V.

*p*5: statistical significance between group V versus groupVI.

Test of significance *p* < 0.05.

SD: Standard deviation.

### Electron microscopy

**Figure 4.**
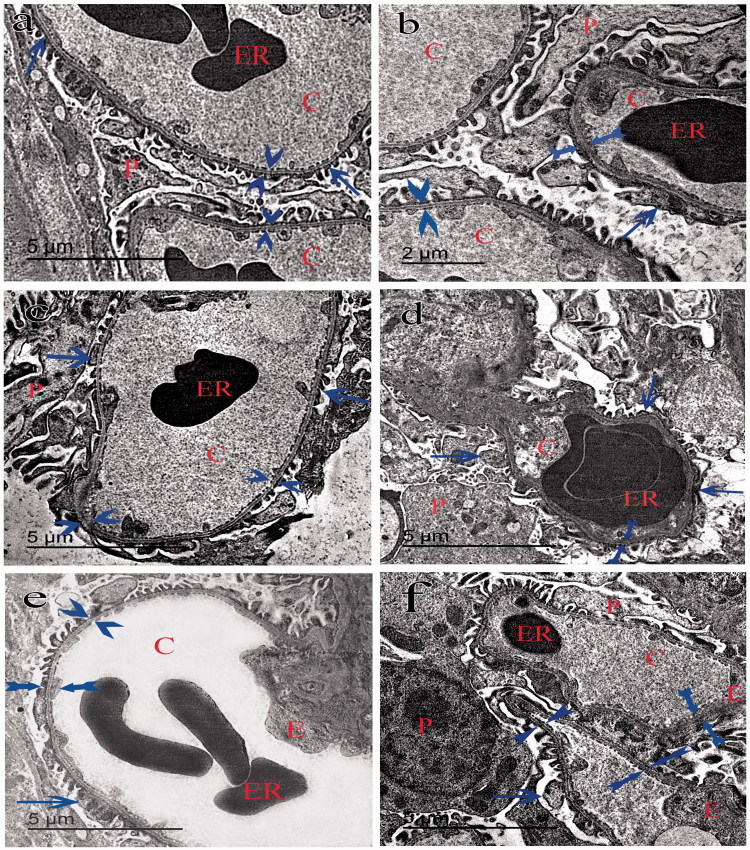
Transmission electron micrograph of the glomerulus: showing typical glomerular capillaries (C) lined with endothelial cells (E) containing erythrocytes (ER) in all groups with uniform thickness of basement membrane lamina densa (arrow-heads) in groups I (control group) (a) and III (estradiol treated group)(c). Areas of uniform thickening (arrow-heads) and areas of irregular thickening of the basement membrane (tailed arrows) in groups II (ovarectomized group) (b), V (estradiol and tamoxifen treated group) (e) and VI (tamoxifen and estradiol treated group) (f) is clear. Areas of irregular thickening of the basement membrane (tailed arrows) in group IV (tamoxifen treated group) is noticed. The foot processes (arrows) of the podocytes (P) are regularly distributed with almost uniform filtrum slits in groups I and III and irregularly distributed with abnormal filtrum slits in groups II, IV, V and VI.

**Figure 5.**
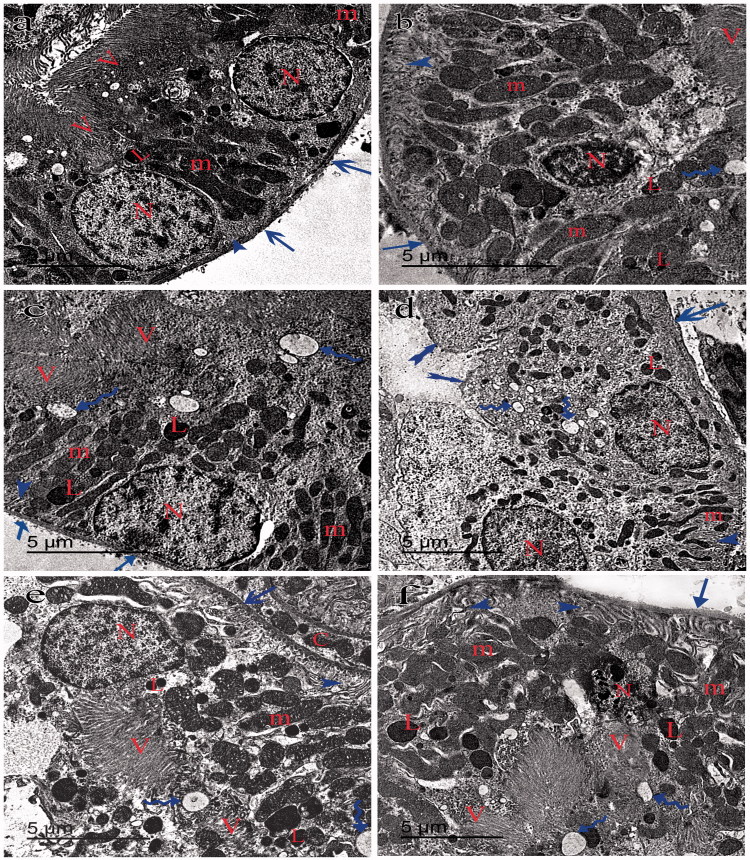
Transmission electron micrograph of the PT showing the lining cells of proximal tubules of the group I (a) with a uniform thickness of basement membrane (arrows) and rounded nucleus close to the base of the cell (N). The mitochondria (m) appears swollen and bizarre shaped in groups II (b), IV (d) and VI (f) and elongated in group III (c) and V (e). Lysosomes (L) were more numerous in groups II and IV. Regular basal enfolding (arrow-head) and apical microvilli (V) in groups I and III. Ill-defined basal enfolding (arrow-heads), cytoplasmic vacuoles (curved arrow) and pyknotic nucleus (N) away from basement membrane (arrows) in groups II, IV, V and VI can be identified.

**Figure 6.**
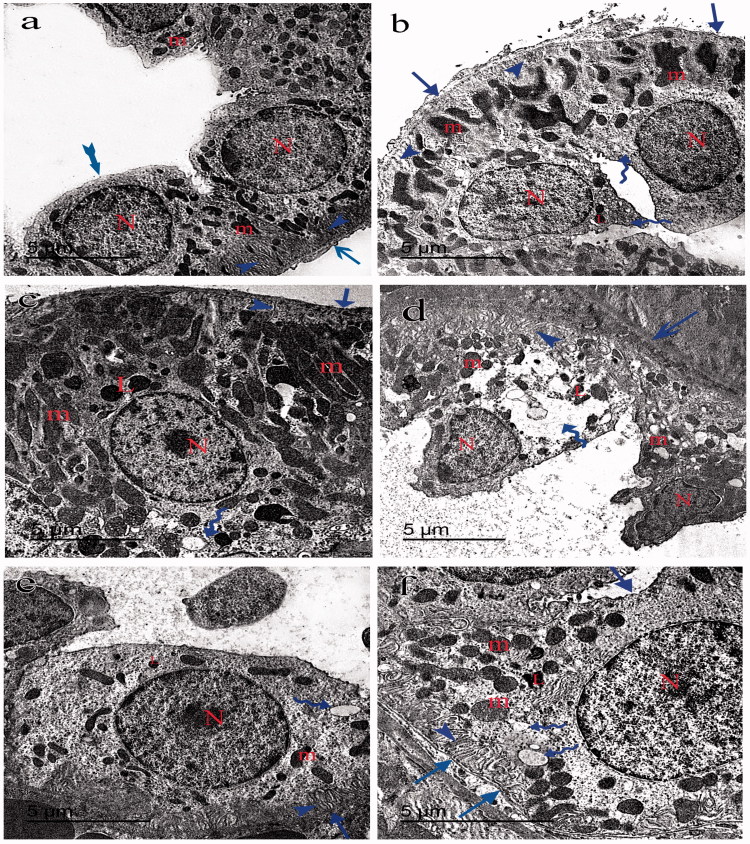
Transmission electron micrograph of the DT showing the lining cells with definite cell border (tailed arrow) in group I (a). The lining cells rest on an irregular basal lamina (arrows) in groups II (b), III (c), IV (d), V (e) and VI (f). The nucleus (N) appears pyknotic in groups II and IV and rounded in groups I, III, V and VI. The mitochondria (m) is elongated in groups I, III and V and pizzar-shaped in groups II, IV and VI. The basal enfolding (arrow-heads) are regular in groups I and III and ill-defined with cytoplasmic vacuoles (curved arrow) in groups II, IV and VI. Few cytoplasmic vacuoles (curved arrows) in group V can be seen.

Transmission electron microscopy of group I showed typical glomerular capillaries with uniform thickened basement membrane and typical lining cells of PT and DT (Figures. 4(a), 5(a) & 6(a)). Group II showed irregular thickening of the basement membrane of the glomeruli. The foot processes of the podocytes appeared irregularly distributed with abnormal spaced filtrum slits (Figure 4(b)). The PT had retained some of the free border microvilli but had swollen mitochondria, numerous secondary lysosomes, cytoplasmic vacuoles and pyknotic nuclei (Figure 5(b)). The DT had elongated bizzare-shaped mitochondria and cytoplasmic vacuoles (Figure 6(b)). Group III showed almost typical glomeruli (Figure 4(c)). The PT and DT had few cytoplasmic vacuoles (Figures 5(c) & 6(c)). Group IV had the glomerular capillaries packed with erythrocytes with areas of irregular thickening of the basement membrane and abnormal foot processes of the podocytes (Figure 4(d)). The PT had fewer microvilli, swollen mitochondria, numerous secondary lysosomes and cytoplasmic vacuoles (Figure 5(d)). DT showed irregular thick basal lamina with scarce bizzare-shaped mitochondria, cytoplasmic vacuoles, and pyknotic nuclei (Figure 6(d)). Group V glomeruli had few areas of irregular thickening of the basement membrane and abnormally distributed filtrum slits (Figure 6(e)). The PT had detached microvilli, elongated swollen mitochondria, secondary lysosomes and cytoplasmic vacuoles (Figure 5(e)). DT had apparent normal elongated mitochondria with some cytoplasmic vacuoles (Figure 6(e)). Group VI glomerular capillaries, PT and DT are more or less close to that of group V (Figures 4(f), 5(f) & 6(f)).

## Discussion

The endocrine functions of the kidney represent one of the important aspects of hormone physiology and pathology. Though, this subject does not seem to have received due attention in the literature [50]. Estradiol is a fundamental hormone in the maintenance of the functions of the renal tissue. It is feasible that the abrupt reduction in E2 levels by OVX can trigger complex functional and structural disturbances with consequent changes of excretory and endocrine functions of the kidney. Serum creatinine and BUN are measured as an index of renal function. A significant increase in their levels with variable values was in all the experimental groups following OVX. The highest increase in their levels was in group IV when compared with the group I. The increased level of SCr in group II (OVX alone group) can be explained either by increased SCr production by muscles as a result of absence of estrogen anabolic effect [51] or by the decrease SCr clearance by the kidneys due to decreased renal perfusion and GFR induced by oxidative stress mediated by OVX [52].

At the same time, OVX significantly increased MDA and decreased GPx, CAT, and SOD. These findings of oxidative stress are accompanied by the morphological alteration in the form of glomerular hypertrophy and narrowing of the Bowman’s space with degenerative changes of the PT epithelial cells. The ultrastructural examination of the glomeruli showed areas of irregular thickening of the glomerular basement membrane with irregularly distributed abnormal filtrum slits of podocytes. PT cell lining showed numerous secondary lysosomes, cytoplasmic vacuoles and pyknotic nuclei. The increase in lysosomes was explained by the increased autophagic process and decreased activity of free ribosome, endoplasmic reticulum and Golgi apparatus secondary to oxidative stress [53].

The significant decrease in oxidative parameters was observed in E2 supplemented groups (III, V & VI). These results were in agreement with the observations made by Azarkish et al. [54]. Ovariectomy effects were explained by an increase in oxidative stress parameter (MDA) and decreased in the levels of antioxidants [55]. Estradiol supplementation alleviated this renal tissue stress through its effect on glomerular mesangial cells and the smooth muscles cells of blood vessels by activating the expression of anti-inflammatory cytokines and interleukine-6 [56]. Several previous studies have attributed the protective effects of E2 to nitric oxide synthases system [57], antioxidant [58], and proinflammatory properties response [59].

Tamoxifen-induced nephrotoxicity was approved by the increase of BUN and SCr levels in group IV, when compared with group II. While this TAM-induced nephrotoxicity was not marked in E2-treated groups (III & V). The decreased level of SCr and BUN in group VI in comparison with group V can be explained by the blocking effect of ERs by TAM and increased TAM-induced PGE2 and EPO synthesis.

At the same time, TAM significantly increased the serum level of MDA and significantly decreased GPx, CAT, and SOD in groups IV & VI. The E2 antioxidant action was less notifiable in group VI, simply because the prior use of TAM minimized this effect. A less discernible effect was detected in group V when E2 was used before TAM.

In OVX females treated with TAM, E2 reversed the levels of MDA, GPx, CAT, and SOD. This can be explained by the ability of TAM or its metabolites to bind to the ERs masking its action and covalently bind to DNA leading to the formation of DNA adduct [30]. Tamoxifen induces oxidative stress and overproduction of ROS with an increase in lipid peroxidation which may be associated with TAM-induced release of iron ions which enhances the generation of hydroxyl radicals [29]. These changes cause renal vasoconstriction with altered glomerular filtration rate which may explain the observed increase in the kidney function biomarkers (SCr and BUN). The findings support the previous observations that compounds with antioxidant properties as E2 can alleviate the TAM-induced nephrotoxicity was developed [60]. This could be further explained by our experience of administration of E2 in group III and V significantly improved the levels of BUN and SCr, elevated the antioxidant enzymes and attenuated the MDA. This result was explained by the fact that E2 inhibits lipid and protein oxidation, preserves membrane permeability, and reduces the level of hydroxyl radicals [29] by its polyphenolic compounds [61].

The extensive changes either in light microscopy or in the ultrastructural examination in groups IV and VI were attributed to vasoactive mediators induced by oxidative stress [29,49]. The protective effect of E2 was approved histopathologically by minimal glomerular, PT and DT changes when compared to the OVX rats. This is in agreement with the previous studies which reported the same findings and attributed the changes to the enhancement of cytoplasmic anabolism by substitution of E2 [53].

Ovariectomy induced a significant increase in EPO level in group II. This elevated level occurred secondary to its augmented production from interstitial cells of the renal cortex, near the base of the PT [4]. Tamoxifen increased EPO level in group IV by its EPO-stimulating ability of renal peritubular cells [62]. Group III and V showed significant change while group VI had a non-significant change. This effect was attributed to effects of E2 on EPO via inhibiting renal EPO gene expression which is mediated by increasing nitric oxide production [63,64]. Furthermore, EPO itself was found to act as an antioxidant and antiapoptotic agent and has a protective effect against OVX & TAM-induced nephrotoxicity [65].

Our results revealed that OVX induced a significant increase in PR that was more with TAM in groups IV. In accordance with our findings that OVX increased PR, some studies reported its increase post-menopause [66]. Reduction in E2 activates RAS and increases the expression of endothelin-1 which is a known vasoconstrictive agent that causes inflammation and oxidative stress with subsequent acute renal function impairment, associated with mesangial cell proliferation and inflammation [67]. By light microscopy, the macula densa appeared more deeply stained in TAM-treated group indicating its hyperactivity. Our results showed that PT retained the free border microvilli and production of angiotensin converting enzyme explaining the associated increase in MDA as a result of Ang II.

The elevation in the levels of PR due to TAM-induced toxicity can be explained by the decrease in the glomerular filtration rate. This finding is in concordance with Miller et al. [68]. Moreover, our results showed that E2 supplementation decreased PR in group III. This result was explained by Pasqualini et al. [69] suggesting that E2 increases angiotensin concentrations by increasing PRS or by enhancing angiotensin synthesis. By negative feedback mechanism, Ang II might exert a direct renal inhibitory effect on renin release or indirect inhibition could result from the increased plasma volume as a result of the effect of Ang II and aldosterone on the DT [70]. Our results are in agreement with the hypothesis that E2 does not stimulate the synthesis and release of renin by the kidney [71]. The response to E2 is confirmed by the apparent normal appearance of the DT in the ultrastructural examination.

Our study revealed that OVX induced a significant increase of PGE2 that is more evident with the TAM (group VI). In addition to this, there was a significant increase of PGE2 in E2 treated groups (III & V).

Previous studies have demonstrated that the PGE2 formed by COX-2 in the renal cortex participates in the control of renin synthesis [9] and that its expression is regulated by dietary salt intake [72] and Ang II [73]. However, the effects of OVX on expression and function of renal COX-2 is still unclear. The present study revealed a significant increase of PGE2 as a result of OVX which aggravated further after administration of E2. This finding was explained by that OVX enhanced the expression of COX-2 in the interstitial cells adjacent to the macula densa through proinflammatory mediators and oxidative stress mechanisms that is independent to salt intake. This was associated with increased urinary excretion of PGE2 in rats [74]. Therefore, E2-dependent COX-2 expression plays an important role in the regulation of PGE2 level and the changes produced by it in regards to renal blood flow and the protective mechanisms in kidneys against various stress conditions. A study by Pedram et al. [73], revealed that the COX-2 promoter gene contains an E2-binding site suggesting that E2 may directly affect the expression of the COX-2.

Our results showed a significant increase in PGE2 by E2 (groups III, V, VI). This is corresponding with the previous studies which demonstrated that E2 influence renal COX-2 through stimulation of the nitric oxide systems by up-regulation of neuronal nitric oxide synthesis [75]. A study by Armando et al. [76] showed that E2 up-regulates renal Ang II type 2 receptors which antagonize the inhibitory effect of Ang II type 1 receptors on Ang II synthesis that stimulates renal COX-2 expression increasing PGE2 [77]. TAM-induced PGE2 increase can be attributed to the direct stimulatory effect of TAM on COX2 expression [78]. Ovariectomy involves both mediators; nitric oxide and Ang II in the up-regulation of renal COX-2.

Our data showed that there was a significant decrease in SD in OVX group (II) and TAM-treated groups (IV & VI) and a significant increase in SD in E2 treated groups (III &V). The mitochondria of cell lining of PT of groups II and IV appeared swollen and bizarre shaped which might cause the decrease SD level in these groups. Estradiol administration changed the mitochondrial shape in the PT of group III and V to become longitudinal and mildly swollen. It is observed that TAM decreases the synthesis of the SD via inhibition of expression of 1-hydroxylase enzyme [79], hence vitamin D3 supplementation is advised during treatment of breast cancer by TAM to compensate for its deficiency and antiproliferative effects.

In the present study, the level of plasma estradiol was measured to monitor E2 levels during the experimental study. As E2 and TAM actions are mediated by ERs, ER-β stain was used to demonstrate this action. Our results showed a progressive increase in ER-β stain expression in the absence of E2 (groups II, IV and VI). On the other hand, a restoration of the weak expression accompanied E2 supplementation (groups III and V). The dynamic balance between ER-β and ER-α is important to mediate the proper E2 action [25,80]. The ER-β has been shown to be specifically important in the inflammatory response to OVX [81]. The E2 signaling of ER-β has a protective effect on the renal tissue by mediating vascular relaxation [56]. The increase in ER-β occurs as an up-regulation mechanism as reported in previous studies [25]. On the contrary, Rogers et al. [82] reported that there is no effect of OVX on ER-β expression. This dissociation was explained by possible genetic differences in the animal strains used [25].

Our study showed that E2 replacement ameliorated TAM-induced nephrotoxicity and oxidative stress. The improvement of renal function and structural changes was explained by E2 membrane signaling through binding to ER receptors initiating transcriptional regulation of the cell function [23]. A better understanding of ERs-E2 signaling pathway is in need of immense investigations.

## Conclusion

The present study supports the concept that E2 has a protective effect against renal structural changes and endocrine dysfunctions induced by OVX and/or TAM. The depletion of the renal redox system together with the liberation of free radicals in response to E2 deficiency or TAM is blamed for the accompanying renal injury. Estradiol improves TAM-induced renal injury possibly by its antioxidant properties. ER-β plays an important role in mediating the actions of E2 and its antagonists. Further investigations are needed to disclose the knowledge about the expression level of ERs and their exact role in mediating E2 and TAM actions.
